# The Immune System Throws Its Traps: Cells and Their Extracellular Traps in Disease and Protection

**DOI:** 10.3390/cells10081891

**Published:** 2021-07-26

**Authors:** Fátima Conceição-Silva, Clarissa S. M. Reis, Paula Mello De Luca, Jessica Leite-Silva, Marta A. Santiago, Alexandre Morrot, Fernanda N. Morgado

**Affiliations:** 1 Laboratory of Immunoparasitology, Oswaldo Cruz Institute (IOC), Fundação Oswaldo Cruz (Fiocruz), Rio de Janeiro 21.040-360, RJ, Brazil; clarissamotareis@gmail.com (C.S.M.R.); pmdeluca@ioc.fiocruz.br (P.M.D.L.); jessicaleite-@hotmail.com (J.L.-S.); marta.santiago@ioc.fiocruz.br (M.A.S.); alexandre.morrot@ioc.fiocruz.br (A.M.); morgado@ioc.fiocruz.br (F.N.M.); 2 Postgraduate Program in Clinical Research in Infectious Diseases, INI-Fiocruz, Rio de Janeiro 21.040-360, RJ, Brazil; 3 Postgraduate Program in Parasitic Biology, IOC-Fiocruz, Rio de Janeiro 21.040-360, RJ, Brazil; 4 Tuberculosis Research Laboratory, Faculty of Medicine, Federal University of Rio de Janeiro-RJ, Rio de Janeiro 21.941-901, RJ, Brazil

**Keywords:** extracellular traps (ETs), neutrophils ETs, macrophage ETs, mast cell ETs, eosinophil ETs, lymphocyte ETs, basophil ETs, dendritic cell ETs, COVID-19

## Abstract

The first formal description of the microbicidal activity of extracellular traps (ETs) containing DNA occurred in neutrophils in 2004. Since then, ETs have been identified in different populations of cells involved in both innate and adaptive immune responses. Much of the knowledge has been obtained from in vitro or ex vivo studies; however, in vivo evaluations in experimental models and human biological materials have corroborated some of the results obtained. Two types of ETs have been described—suicidal and vital ETs, with or without the death of the producer cell. The studies showed that the same cell type may have more than one ETs formation mechanism and that different cells may have similar ETs formation mechanisms. ETs can act by controlling or promoting the mechanisms involved in the development and evolution of various infectious and non-infectious diseases, such as autoimmune, cardiovascular, thrombotic, and neoplastic diseases, among others. This review discusses the presence of ETs in neutrophils, macrophages, mast cells, eosinophils, basophils, plasmacytoid dendritic cells, and recent evidence of the presence of ETs in B lymphocytes, CD4+ T lymphocytes, and CD8+ T lymphocytes. Moreover, due to recently collected information, the effect of ETs on COVID-19 is also discussed.

## 1. Preamble

With the great impetus given to the understanding of cellular functions in the immune system in the early 1950s, much information has been obtained; nevertheless, some mechanisms have not yet been fully elucidated. This knowledge is crucial to understand the mechanisms of disease and/or protection, since knowing how to recognize in what way diseases occur develop new treatments and effective vaccines with fewer adverse effects. Thus, paraphrasing Stephen Hawking [1], cells are a universe involved in a plasma membrane, and new components and functions are described every day. The formation of extracellular traps (ETs) containing DNA is clear evidence. The mechanisms described are varied and can be identified as inhibitors or facilitators of lesions, and some recent publications describe details of the ETs formation, their mechanisms, and structure [2,3,4,5]. The ETs formation seems to be a fast event, and perhaps, for this reason, it has not been easily observed in vivo until now. In addition, some cells in which their formation has been described are difficult to handle. Thus, much of the knowledge was obtained from in vitro or ex vivo studies with different protocols (reviewed in [2]). However, evaluations in experimental models and human samples have corroborated many of these results [6]. Although some mechanisms and effects of ETs release still need further elucidation, what is already known shows the importance of ETs in the control and/or development of the immune response.

## 2. General Background

The term ETosis was coined in the late 2000s [7,8] to designate a type of cell death promoted by decondensation of nucleic DNA called ETs. Based on the first description of the microbicidal activity of neutrophils [9], several studies showed that ETs play a pivotal role in infection control through an innate immune response, and their understanding, scope, and particularities have been described over subsequent years. Additionally, what has been previously described as having a beneficial action upon the resolution of inflammation is now known to be also capable of expanding inflammatory processes. It is also involved in the pathogenesis of various infectious and non-infectious diseases, such as autoimmune, cardiovascular, thrombotic, and neoplastic diseases, etc. [2,10,11,12,13]. It is known that DNA must be removed from the system rapidly, since it may stimulate an inflammatory response, as observed in the suggested relationship between the presence of extracellular DNA and several autoimmune diseases, such as systemic lupus erythematosus (SLE) and rheumatoid arthritis (RA), as well as HIV infection, cardiovascular diseases, and neoplasms [14,15,16,17].

Besides neutrophils, ETs formation has already been identified in macrophages, mast cells, eosinophils, basophils, plasmacytoid dendritic cells, and recently in lymphocytes. Hence, the term originally coined (NETs—neutrophil extracellular traps) was adapted according to the cell type involved. The cell death resulting from this process was called ETosis to differentiate it from other types of cell death, such as necrosis, apoptosis, necroptosis, and pyroptosis [7,8,13,18].

It is worth noting that recent studies suggest that the intensity and type of inflammatory response produced by DNA traps depend on the type of producing cell, the origin of the DNA involved, and on the presence of other associated products or not with DNA, such as enzymes, plasma proteins, histones, etc. Lately, it has been found that not all ETs formation produces cell death [19,20,21], and a new classification of DNA types released into the extracellular environment has been organized [2,21]. In summary, the previously detected types of DNA release are divided into: (1) Suicidal ETosis—nuclear DNA release with histones, which occurs 3–8 h after cell activation. The nuclear chromatin is decondensed, and after expanding into the cytoplasm, it associates with cytoplasmic and granular proteins. As expansion continues, the plasma membrane breaks, causing the death of the involved cell and the release of the DNA associated with histones and other proteins into the extracellular environment, thus forming filament traps in continuous expansion. This can be detected after stimulation by phorbol myristate acetate (PMA), concanavalin A (ConA), interferon (IFN), infectious agents, immune complexes, and autoantibodies, among others [2,10]; (2) vital ETosis—release of DNA, maintaining cell viability. The vital ETs formation is an early/rapid process that usually occurs between 5–60 min after cell activation, which continues to perform its functions, such as chemotaxis and phagocytosis after externalization of DNA (nuclear or mitochondrial) simultaneously as degranulation and protein release [2,19,20,21,22]. Two main forms have been described: (1) Extracellular environment release of vesicles containing nuclear DNA, which expands to form extracellular traps. Initially, it maintains cell viability and functions as chemotaxis, adherence, and phagocytosis. It has been well described for neutrophils, even when anuclear cells are identified, since the granules and outer membrane are preserved [19,20,21]. In vitro studies suggest a rapid process when compared to ETs produced by suicidal ETosis. It has already been described that later, these cells also die and are usually phagocytosed by macrophages; (2) produced from mitochondrial DNA (mtDNA) in a mechanism dependent on reactive oxygen species (ROS), which is also related to the maintenance of cell viability. Essentially, two forms of mtDNA exteriorization are proposed: (A) Initial release into cytosol and thereafter into vesicles that merge with the plasma membrane, exteriorizing their content; and (B) mitochondrial and plasma membrane fusion, producing the direct release of DNA content, into the extracellular environment. However, the latter has not yet been fully proven [2,3,4,5,10,13,23,24].

**Figure 1:**
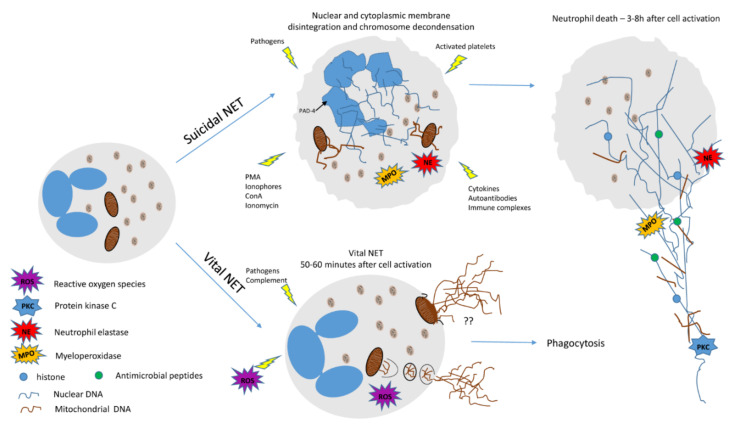
Scheme showing the formation of suicidal and vital NETs. Suicidal NETs: Occurs 3–8 h after cell activation and ends with the death of neutrophils. It starts with chromosomal decondensation and nuclear membrane disintegration, followed by decondensation and release of mitochondrial DNA to the cytosol. Finally, the cytoplasmic membrane disintegrates, releasing nuclear and mitochondrial DNA, as well as granular content into the extracellular environment. Vital NETs: Occurs 50–60 min after cell activation maintaining neutrophil viability and phagocytosis. Two forms of exteriorization of mitochondrial DNA are proposed: a-Initial release into the cytosol and thereafter into vesicles that merge with the plasma membrane, exteriorizing their content; and b-fusion of the mitochondrial and plasma membrane, producing the direct release of the content of DNA into the extracellular environment. However, the latter has not yet been fully proven. Various stimuli for the formation of suicidal and vital NETs have already been described, the most commonly seen in the figure. Some variations related to stimuli and composition can occur with ETs produced by other types of cells.

Data collection has shown that the same cell type can present more than one ETs formation mechanism simultaneously or consecutively, and that different cell types can present similar ETs formation mechanisms [2,14,25,26]. Nevertheless, further studies are needed to better understand the relationship between the origin of ETs formation and its role in cell viability and in the immune response. Figure 1 shows a scheme of the different types of ETs produced by neutrophils.

Concerning infectious diseases, releasing ETs into the extracellular environment promotes the capture and death of the surrounding microorganisms [27,28,29,30,31]. Moreover, the presence of DNA and other proteins can lead to an increment in local inflammation, including exacerbation of the disease [10,32]. Even so, it has been described that the evasion of microorganisms by DNase production, the inhibition of cell recruitment from the immune response, as well as the evasion of bacteria by modification of the cell wall with alteration of the ionic charge, hampers the coupling of the microorganism to ETs (reviewed by the authors of [33]).

In terms of location, ETs can be virtually seen in any compartment of the human body, such as solid organs and blood, where clusters of ETs are cell-free and may be involved in the formation of thrombus and tissue injury [5,24]. On the other hand, the presence of enzymes can degrade inflammatory mediators, which could lead to a decrease in the inflammatory process, promoting the resolution of lesions. We will discuss the presence of ETs in neutrophils, macrophages, mast cells, eosinophils, basophils, plasmacytoid dendritic cells, and new evidence of the presence of ETs in B lymphocytes, CD4+ T lymphocytes, and CD8+ T lymphocytes. Moreover, due to recent data, the effect of ETs on COVID-19 will also be discussed.

## 3. Neutrophils

Neutrophils are the first cells attracted to the site of tissue injury. They are polymorphonuclear leukocytes originated from a myeloid progenitor in the bone marrow, and present nucleic acid organized in a multilobulated form containing three to five lobes, connected by chromatin. Indeed, about 60% of the white blood cells produced by bone marrow are neutrophils, although this number may change depending on the stimulus [34,35]. Though there is a plethora of neutrophils, they have a fairly short lifespan, and in the absence of signs of infection or inflammation, they die 6 to 8 h later via a programmed cell death process [36]. They are removed from the tissues by macrophages, preventing the release of their potentially harmful content into the tissues [37]. The maintenance of neutrophil cell debris in tissues has been associated with developing diseases, such as cystic fibrosis, chronic obstructive pulmonary disease (COPD), and RA [38].

Neutrophils act upon the innate immune response with inflammatory responses against pathogens (protozoa, bacteria, fungi, viruses) via intra- and extracellular mechanisms, such as phagocytosis, secretion of granular enzymes, ROS production, and NETs formation [9,39,40]. After the onset of the early stage of tissue injury, neutrophils rapidly migrate into tissues through the expression of chemotactic factors and adhesion molecules (P-selectin and E-selectin) expressed in endothelial cells [41,42]. Thus, neutrophils express the chemokine receptors CXCR1 and CXCR2 that bind to CXCL1 and CXCL8; the main chemokine that maintains migration of neutrophils into the affected tissue [43,44]. Classically, within the tissues, neutrophils initiate the process of phagocytosis, which can occur through recognizing PAMP (Pathogen-Associated Molecular Patterns), through TLR (Toll-like Receptors), or through opsonization and connection with Fc receptors, complement receptors (CR1 and CR3), and C-type lectins. The pathogen is then destroyed by the enzymes present in its granules and by the formation of ROS [39,40]. These granules are classified into azurophils (or primary), specific (or secondary), and gelatinase (or tertiary). Azurophilic granules consist of myeloperoxidase (MPO), defensins, lysozymes, and antibacterial proteins with serine protease activity (NE-neutrophil elastase, Proteinase 3, and cathepsin G) [45,46]. However, specific granules have lactoferrin and lysozymes, and gelatinase granules consist of very few antimicrobial substances, which function as storage for metalloproteases (gelatinase and leucolysin) [38,47].

During the pathogen internalization/neutrophil activation process, Nicotinamide Adenine Dinucleotide Phosphate (NADPH) oxidase is activated by converting molecular oxygen into superoxide anion, hydrogen peroxide (H_2_O_2_), and free radicals (such as ROS) [35,48] in the oxidative burst with active participation in the elimination of pathogens. Furthermore, the formation of hypochlorous acid (HOCl) occurs through the catalysis of hydrogen peroxide [49].

In 2004, Brinkmann et al. described the control of aggressive agents by neutrophils stemming from the decondensation and release of DNA called NETs. The release of nuclear DNA by neutrophils had been previously verified by Takei et al. and described as a new form of cell death called NETosis [9,50]. Subsequently, several studies have analyzed their formation mechanisms and function [5,10,22].

Typically, NETs are 3D structures composed of DNA, histones (H1, H2A, H2B, H3, and H4), proteins of three types of granules, such as NE, MPO, cathepsin G, leukocyte proteinase 3 (PR3), azurocidin, lysozyme C, and antimicrobial peptides, i.e., defensins and cathelicidins, that act as secreted physical barriers to restrain the spread of free pathogens present in the extracellular environment or that evaded phagosomes [2,3,5]. The mechanism of NETs formation can be induced by several stimuli: Microbial (bacteria, fungi, protozoa, viruses) [3,23], immune complexes [6,51], cytokines [6,51], damage-associated molecular patterns (DAMPs) [52], activated platelets [20,21], microcrystals (cholesterol, calcium carbonate) [6,23,53], among others. The phorbol ester (mainly PMA) and ionophores (A23187, nigericin) are important inducers of NETs in vitro [4,5,23] (Figure 1).

Although ETs are widely studied, NETs are a complex phenomenon, and there are still questions to be elucidated about the mechanisms involved in their formation (origin of the DNA, programmed cell death, signaling pathways), their role in host defense, and the pathophysiology of some diseases [6]. Many studies have been carried out in recent years; however, factors, such as the origin of the neutrophils used in the experiments, the isolation methods, the culture media, and/or the cell reactivation may influence the results, making it difficult to compare the results and the outline of unified knowledge about NETs [54,55]. Despite variations in nomenclature and classification, the mechanisms can share pathways, resulting in the release of extracellular DNA [2,5,10,20,22,23,55]. In order to standardize, the Nomenclature Committee on Cell Death (NCCD) recommends that the term “NETosis” be replaced by NETs formation, since NETs can be produced without cell death [21,56].

**Figure 2:**
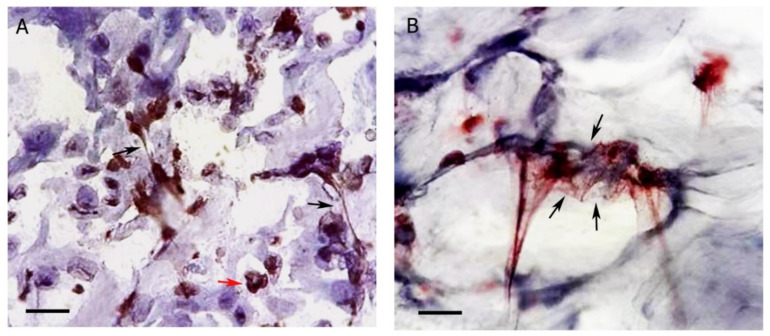
Neutrophils and NETs in skin lesions of patients with Cutaneous Leishmaniasis. Neutrophil elastase was used as a marker of neutrophils and NETs by immunohistochemistry (brown staining—aminoethyl carbazole—AEC). (**A**) Apparent whole neutrophils (red arrows) and NETs with a spiky aspect (black arrows). (**B**) Aggregated NETs (black arrows). Magnification bar A = 25 μm and magnification bar B = 10 μm. Counterstaining was carried out using Meyer’s hematoxylin.

Morphologically, several types of NETs have been described, roughly dense, wider, or more delicate, isolated, or forming aggregates, etc., and can be organized according to the stimulus, pH, neutrophil concentration, and pathogens present at the site of NETs formation [24]. For example, tapering formations called spike NETs can be induced by the complement factor C5a (C5a), among other stimuli. However, the alkaline pH, commonly observed in chronic wounds, favors the formation of cloudy NETs and giant bicarbonate-induced aggregated NETs (aggNETs), which can reduce necrotic areas [22,24,57]. AggNETs are large cloudy or clumpy traps that form in places with high-density neutrophils and contain viable neutrophils, microorganisms, and enzymes. They act upon the elimination of pathogens and the degradation of inflammatory mediators, favoring healing. However, they can also cause vessel and duct obstruction, due to their size and sticky nature [24,58,59]. The formation of NETs with different morphology can be observed in active cutaneous lesions of American Tegumentary Leishmaniasis (ATL) [29] (Figure 2).

The cellular pathways involved in NETs formation are complex, and their protein composition seems to be stimulus-dependent [10,55]. Several enzymes and signaling proteins, such as protein kinase C (PKC), extracellular signal-regulated kinase (ERK), MEK (MAPK/ERK kinase), participate in the process. NE and MPO play a critical role in histone cleavage and inactivation, chromatin decondensation, and nuclear membrane degradation, enabling the combination of nuclear and cytoplasmic contents. Protein-arginine deiminase type 4 (PAD-4) migrates to the nucleus of neutrophils to induce an increase in the permeability of the nuclear membrane and also takes part in the chromatin decondensation process through the citrullination of histones. Despite being essential in the NETs formation, there is still no clear consensus on whether its role is fundamental in suicidal NETs formation [5,10,22,55].

NETs can also be classified as NADPH (NOX)-dependent and NOX-independent according to ROS production, since the presence or absence of ROS produced by NADPH oxidase in the cytoplasm or mitochondria seems to influence their formation [3,4,5,22,23,54]. In the formation of NOX-dependent NETs, various stimuli (e.g., PMA, cholesterol crystals, fungi, bacteria) induce ROS production by NADPH oxidase [10,23,54,55]. It has been found that NOX-independent NETs formation can be induced by calcium ionophores (e.g., A23128, A23187, ionomycin), uric acid crystals, nicotine, and immune complexes [22,25,55,60], but it is still questionable whether these mechanisms are ROS-independent, or whether mitochondrial ROS (mtROS) are produced [54]. Vital NETs produced from mtDNA seem to depend on mtROS [61,62,63]. On the other hand, vital NETs with nuclear DNA seem to be ROS-independent [19,20,21]. Moreover, it has been reported that *Candida albicans*, gram-positive bacteria Group B *Streptococcus*, and *Leishmania amazonensis* may induce the formation of these traps with little or no ROS production by neutrophils, possibly because these pathogens can generate their own ROS [4,64].

In candidiasis, neutrophils are the major cells recruited to destroy yeasts and hyphae of the fungus, although the latter form cannot be phagocyted, due to its size. However, hyphae are destroyed by the NETs formation even independently of opsonization, which may occur in both hyphae and yeast. Thus, the elimination of these fungi results from the activity of the granules, mainly calprotectin. This process depends on recognizing β-glucan by CR3, fibronectin, and ERK, but is ROS-independent and the NETs formed are classified as vital [65]. Though, *Aspergillus fumigatus*, a fungus that causes aspergillosis, an opportunistic disease that can lead to severe lung involvement, stimulates the NETs formation from β-glucan in a ROS-dependent process. However, it has been demonstrated that these NETs cannot kill the fungus but can prevent its spread and growth [66]. The presence of NETs in active sporotrichosis lesions caused by *Sporothrix schenckii* in both fixed and sporotrichoid forms has also been demonstrated [30].

Infections by Protozoa of the genus *Leishmania*, on the other hand, may stimulate NETs dependent on NE activity, but independent of ROS from NADPH oxidase and PAD-4. NETs have been described as having a protective function against this protozoosis, being able to capture and destroy parasites, except *Leishmania mexicana*, which can escape from this mechanism. Neutrophils have a close relationship with *Leishmania* spp. from the early stages of infection, as they are rapidly recruited into the skin after the entry of the protozoan. Interestingly, it has already been demonstrated that the saliva of *Lutzomyia longipalpis*, one of the insects that transmit this parasite, has endonucleases capable of degrading NETs, which could indirectly act on the pathogenesis of the disease [64,67,68,69,70]. In addition, NETs are also observed in ATL lesions presenting different evolution times, suggesting a continuous role of neutrophils in tissue inflammation [29]. In recent work, da Fonseca-Martins et al. demonstrated that protozoa of the genus *Leihmania* may increase the expression of programmed death ligand-1 (PD-L1) by neutrophils, in favor of their survival, with a consequent suppressor effect associated with progression of the lesion [71].

The protective role of NETs in innate immunity is associated with the resolution of inflammation and healing, along with antimicrobial activities. Nevertheless, if there is no balance between their formation/degradation, NETs can promote tissue damage and inflammation, implicating the pathophysiology of several diseases [3,24,72]. It is the case of infection by the causative agent of Malaria—*Plasmodium falciparum*—which induces NETs formation independent of ROS, but dependent on the MEK/ERK pathway. The components released by neutrophils during the process can cause tissue damage, mainly in the liver, further aggravating the condition [73]. This imbalance in the formation and extravasation of NETs is also evident in cases of sepsis. Despite having an important role in containing bacteria, the content of neutrophils released into the external environment can increase inflammation, cause thrombosis, and, in the worst case, lead to the failure of several organs [74,75].

NETs may also act as self-antigens and induce an immune response with the production of autoantibodies (e.g., anti-damaged-DNA/RNA ribonucleoprotein antibody immune complexes) and DAMPs capable of activating neutrophils and triggering the formation of new NETs. A vicious cycle is created and may exacerbate inflammation and lead to the development of autoimmune diseases, such as SLE [25,51,76]. In the literature, SLE is a well-reported example of loss of tolerance to self-antigens. In these patients, anti-DNA antibodies may deactivate the DNase enzyme, which cleaves NETs. When NETs are not cleaved, they can become a source of self-antigens, thus stimulating the higher production of anti-DNA antibodies [77].

Activated platelets and neutrophils may contribute to an increased risk of adverse cardiovascular events after acute myocardial infarction [78]. NETs are an important product of the platelet-neutrophil axis and contribute to vascular damage in cardiovascular disease [78]. During transmigration, endothelial cells interact with neutrophils and may stimulate the formation of the NETs within the microcirculation and generate an occlusion, leading to thrombotic diseases. Immune-thrombi formation occurs after contact of activated platelets with polymorphonuclear cells [79]. Many works on sepsis have also demonstrated this phenomenon [24,80,81,82,83]. In addition, the participation of interleukin-1β (IL-1β), a pro-inflammatory cytokine, in the tissue factor (TF) release and NETs formation in atherothrombotic events has also been indicated [84]. IL-1β recruits cells by inducing the expression of adhesion molecules on endothelial cells [85] and has already been suggested as a stimulus for NETs production in abdominal aortic aneurysms [86]. In a murine model, NETs and inflammasomes have been shown to cooperatively contribute to venous thrombosis [87]. The stimulation of neutrophils led to the formation of NETs, which, through their histones, promoted a robust activation of caspase-1 in platelets. Using intravital microscopy, the study showed that NETs were colocalized with caspase-1 and platelets at the site of thrombosis [87].

The interaction of activated platelets and neutrophils as causes of vascular damage is also described in myocardial infarction and in autoimmune processes, such as systemic sclerosis, where the mobility group box 1 (HMGB1) protein induces the formation of NETs mediated by autophagy [79,88].

Neutrophil autophagy is a process already discussed by some authors and seems to be related to the production of NETs in several situations, such as sepsis, gout, and fibrotic processes. Fibrosis occurs because of the activation of fibroblasts by the exteriorized content of neutrophils. The process can be harmful, especially in lung diseases with an inflammatory process, and has the participation of components, such as chromatin, histones, MPO, and IL-17 [89,90].

In tumors, it has been found that NETs-derived granule proteins may contribute to the migration of tumor cells from the primary site to other sites, favoring the formation of metastases [11,12,91]. The type of disease associated with NETs is related to the trap formation site and their degradation mechanisms [24].

### NETs and COVID-19

Recent studies reinforce a body of evidence pointing to the participation of neutrophils and especially NETs in COVID-19 [92,93,94,95,96,97,98,99]. As mentioned above, when NETs are excessively induced in vital organs, such as the lung, they are harmful to the body. Patients with severe cases of COVID-19 are predisposed to thrombosis, which is the obstruction of veins and arteries, due to excessive formation of blood clots [100], which is frequently considered one of the main negative consequences of the formation of NETs. Severely infected patients develop an uncontrolled and damaging inflammatory response to host tissues, similar to those seen in cases of sepsis. This excessive damage has been credited to the participation of neutrophils in the acute phase of infection [101].

Severe COVID-19 is also associated with a cytokine storm, characterized by increased plasma concentrations of various inflammatory mediators. Some of them involved in the regulation of neutrophil activity and the expression of chemoattractants that increase the trafficking of neutrophils. Since NETs can induce macrophages to secrete IL1β, and IL1β enhances NET formation in various diseases [84,86], it is possible that a NET–IL1β loop is activated in severe COVID-19, and can participate in the formation of microthrombi and respiratory decompensation.

The significant increase of neutrophils in the bloodstream is one of the markers of COVID-19 severity, being associated with a higher risk of death. The increase of molecules that indicate the presence of NETs has been detected in the sera of critically ill patients [92,94,95,96,97,98], and increased levels of plasma NETs markers correlated with increased COVID-19 severity [96,97]. NETs formation could also be observed in lung autopsies tissues of COVID-19 patients [94,95,96].

The presence and excessive activation of neutrophils by complement, together with platelets and NET formation, have also been associated with severity in SARS-CoV-2 infection, in which TF plays an important role in the thrombogenic activity [102,103]. The results produced by Skendros et al. suggest that the inhibition of C3 may interrupt neutrophil TF release and prevent complement activation [103]. Morrissey et al. identified a population of low-intensity inflammatory neutrophils in COVID-19 patients. These cells expressed intermediate levels of CD16 (CD16Int), an inflammatory profile, and were associated with platelet activation, spontaneous formation of NETs, increased phagocytic capacity, and cytokine production [104]. Furthermore, neutrophils were the main cells found in bronchoalveolar lavage fluid (BALF) and in peripheral blood, associated with high levels of D-dimer, ferritin, and inflammatory cytokines (such as tumor necrosis factor-TNF and IL-6), suggesting the participation of these cells in coagulopathy, systemic inflammation, and severe acute respiratory syndrome associated with COVID-19 [104].

Neutrophil exposure to serum from severe COVID-19 patients was also shown to induce functionally active NETs [93,99]. This can occur either because of the presence of active viral particles in the serum or the presence of inflammatory factors capable of activating the formation of NETs. Other results further indicated that SARS-CoV-2 alone can directly activate NETs in neutrophils incubated with the virus [92,94], indicating in an unprecedented way that the new coronavirus can stimulate neutrophils to release ROS, together with NETs [92]. Another class of weapon used by defense cells to fight infections, these substances act directly to kill invading microorganisms, and simultaneously, stimulate the formation of NETs in the process of activating the blood coagulation cascade, a hallmark of severe cases of COVID-19 [93,100].

These results alert that the activation of neutrophils to release NETs and ROS is possibly one of the important causes of thrombosis in COVID-19 [92,93,94,95,96]. Therefore, it is possible to apply therapeutic strategies on these targets to avoid as much as possible the formation of vascular thrombosis, a harmful response for patients. Affecting organs, such as lungs, kidneys, heart, and brain, the phenomenon is associated with death by cardiorespiratory failure or multiple organ failure [100]. It is important to note that these works point to a biochemical pathway that may target developing new therapies to combat clot formation. NETs can be attacked by existing drugs in different ways. Among the options that can be evaluated, we can consider the medications used to treat cystic fibrosis, which works by disrupting neutrophilic networks and released antioxidants [105]. Colchicin and Anakinra are other existing drugs that could be used as blockers of the inflammatory loop between NETs and IL1β, with several ongoing clinical trials (ClinicalTrials.gov identifiers (accessed on 25 June 2021): NCT02735707, NCT04322565, NCT04322682, NCT04324021, NCT04326790, NCT04328480, NCT04330638) [106].

Examples of NETs and their role in host defense and disease are described in Table 1.

## 4. Macrophages

Macrophages are leukocytes produced in the bone marrow from myeloid progenitors. They leave the bone marrow as peripheral blood monocytes, and, when located in tissues, differentiate into macrophages. In some tissues, they remain for variable periods, being called resident macrophages, and may receive specific denominations according to the tissue, such as histiocytes in the skin, or Kupffer cells in the liver. Macrophages were initially identified by their phagocytosis ability, which can be easily visualized under an optical microscope. As studies on the immune system advanced, macrophages were found to participate in several stages of the immune response to infectious agents, from the initial stimuli to naive T lymphocytes for their differentiation in activated T cells, subsequently acting upon the effector phase of the immune response with intense secretory and microbicidal activity. Finally, when pathogens are eliminated, macrophages act upon the removal of cell debris in the healing process and return to homeostasis. They are involved in the remodeling of the extracellular matrix, angiogenesis, and stimulation of fibroblasts. Moreover, they act significantly on non-infectious inflammatory processes, secreting mediators, and phagocyting cellular debris, among other functions. Macrophage subpopulations have been described, being M1 and M2 the best-known expression profiles (reviewed by the authors of [133]). In brief, the M1 profile is composed of macrophages, activated by the classical IFN-γ pathway and TLR microbial ligands that can express inflammatory cytokines, the inducible nitric oxide synthase (iNOS) enzyme, and the production of nitric oxide (NO) [134]. The M2 profile, however, is activated via an alternative route. The known stimuli are cytokines IL-13 and IL-4, and this cell can express arginase-1, TGF-β, and IL-10 [134]. Typically, the M2 profile is related to pathogens eliminated by the Th2 cells-mediated immune response. Another function described for M2 cells is to induce healing, as they produce fibroblast growth factors, stimulate collagen synthesis, and angiogenesis [135].

Recently, an additional effector function has been described for macrophages: the capacity to release their DNA content to form extracellular traps called METs (macrophage extracellular traps). METs are mostly composed of DNA and histones, but also of MPO, lysozymes, and citrullinated histones (H4Cit3, CitH3) [136,137,138,139,140,141,142,143]. Macrophages can release both nuclear DNA and mtDNA to form METs, which can be composed only of mtDNA, or the association of mtDNA and nuclear DNA [136]. The stimuli for METs formation described in in vitro studies can be NETs, NE, citrullinated histones, ROS, MPO, PMA, HOCl, IL-8, TNF, and IFN-γ [136,137,139,140,144,145,146]. Several infectious agents were also able to stimulate METs in vitro, as shown in Table 2.

METs have already been described in some inflammatory/infectious conditions. In acute epididymitis caused by *Ureaplasma urealyticum*, *Chlamydia trachomatis*, and *E. coli*, METs and NETs were observed in the semen of patients [142]. Three patterns of ETs formation were observed by macrophages and neutrophils in the semen: (1) Spread ETs—structures in the form of elongated bands of decondensed chromatin associated with antimicrobial proteins and composed of fine fibers 15–17 μm diameter; (2) Diffuse ETs—decondensed extracellular chromatin traps, associated with globular antimicrobial proteins and measuring 15–20 μm in diameter; (3) aggregated ETs—high-density release of ETs forming aggregates. However, METs were mainly diffuse and composed of DNA, histones, H4Cit3, and MPO [142].

A recently published protocol demonstrated the METs formation in vitro from macrophages derived from human monocytes [146]. Macrophages were polarized to the M1 profile, and then METs release was stimulated by inflammatory compounds (PMA, HOCl, IL-8, and TNF) [146]. In an experimental in vitro infection model, *C. albicans* stimulated the METs formation in macrophage cell line J774, peritoneal macrophages, and bone marrow-derived from BALB/c mice [136,137]. The METs formation occurred at the beginning of the assay, increased progressively over time, increasing the yeast: Macrophage ratio, and presenting a significant antimicrobial effect [137]. The authors also show the ability of the fungus to degrade METs—when comparing the ETs formation with live and dead yeasts, greater METs formation after stimulation with dead yeasts was observed. Moreover, they showed that there was arbitrary DNA degradation when cocultivation was performed with live yeasts, and there was no change in the amount of arbitrary DNA when performed with dead yeasts. Subsequently, they suggested that *C. albicans* can degrade METs, describing it as a virulence factor and escape mechanism [137]. However, in the study by Liu et al., METs could not control fungal load, despite restraining *C. albicans* [136]. Possibly, the restraint of these pathogens by METs reduces the probability of spreading through the organism, which would be an effector action that contributes to the control the infection, despite having no direct effect on the fungal load. Interaction studies between neutrophils and macrophages in this infection could elucidate some questions, such as whether the yeasts in METs and NETs could be delivered to the macrophages not involved in the formation of ETs, but involved in phagocytosis, as described by Loureiro et al. [137].

*Eimeria ninakohlyakimovae* also induced the METs formation in vitro from goat monocytes [140]. METs were observed after stimulation with different viable evolutionary forms: Sporozoites, sporocysts, and oocysts and confirmed by the colocalization of DNA, MPO, and histones in ETs. The authors confirmed that ROS stimulated the METs formation, since the traps decreased in the presence of the NADPH-oxidase inhibitor: Diphenylene iodondium (DPI). Despite observing the restraint of this protozoan in METs, no direct toxic effect or in vitro control of the parasitic load was verified [140]. The same was observed in the in vitro infection of bovine monocytes by *Besnoitia besnoiti* and *Eimeria bovis* [139]. In addition to the effect of ROS, the authors also confirmed the role of MPO in the induction of METs using a specific inhibitor. In this study, the impact of METs on the parasitic load was small, with only a 2% reduction in parasite numbers [139].

*Mycobacterium tuberculosis* (Mtb) could also stimulate MET formation by human macrophages [144]. This process occurred in highly parasitized macrophages and was induced by INF-γ, regulated by elastase activity, and required the Mtb ESX-1 secretion system. ESX-1 is a virulence factor of Mtb and encodes a protein secretion system that triggers the cell death pathway independent of caspase-1. In the presence of IFN-γ, there was synergism with ESX-1, leading to the macrophage METs formation [144]. Taken together, the data suggest a possible role of METs in the innate immune response to different infectious agents, since METs in vitro have led to the restraint of pathogens, and, in some models, to control the parasitic load.

In the breast and visceral adipose tissue of obese patients, macrophage infiltrates were often observed surrounding dead adipocytes, forming “crown-shaped structures” (CLS), and the presence of these lesions was associated with elevated levels of inflammatory mediators. The authors argue that obesity-induced inflammation of adipose tissue promotes the METs formation within CLS lesions via PAD-4-mediated hypercitrullination of histones [138]. In a db/db mouse model of diabetes, METs were found in adipose tissue and associated with a deleterious effect on inflammation and insulin resistance [146]. In this study, the authors indicate that silencing the hepcidin gene reduced the recruitment of macrophages and inhibited the METs formation, resulting in decreased inflammation (decreased IL-1β and TNF) and insulin resistance [146]. Hepcidin is a peptide primarily produced by hepatocytes and is the key regulator of iron metabolism. It binds to ferroportin on the surface of macrophages and other cells, and consequently, prevents iron leakage, leading to the accumulation of intracellular iron [148]. Iron accumulation in tissues is related to the remodeling in adipocytes and the accumulation of macrophages, which increases the secretion of inflammatory cytokines and oxidative stress [143]. In a study by Zhang et al., the authors discuss a possible role of hepcidin in regulating the METs formation [143]. Although interesting, further studies are needed to prove whether hepcidin has a direct or indirect effect on the induction of METs.

Published data have shown that, depending on the disease model studied, METs can be beneficial or harmful to the body [136,137,138,141,143]. The balance between the ETs formation and their degradation is essential to prevent infections and inflammatory diseases. Thus, an excessive formation or delayed degradation of ETs may cause tissue damage, due to the toxic components associated with this structure. Therefore, macrophages play a key role in removing ETs from different cell types.

### Cooperation between Macrophages and Neutrophils in the Extracellular Traps Context

Macrophages, besides participating in the inflammatory response, play an essential role in removing cellular debris and toxic products, potentially harmful to the organism, since they can perpetuate the stimulus to inflammation. Cell-free DNA, the main component of ETs, is recognized as DAMP and induces tissue injury [145]. In a diabetes model in mice, it was observed that NETs that promoted inflammation and progression of atherosclerosis were more abundant and prevented the resolution of inflammation during the wound healing process [149]. The NETs+ areas were enriched by NOS2+ macrophages and by the increased activation of inflammasomes, suggesting that NETs exacerbate the inflammation of macrophages and induce their differentiation to the M1 profile. In this study, the use of DNase 1 decreased the number of NETs, due to the degradation of chromatin fibers, thus reducing inflammation and disease severity [149]. In Behcet’s disease, NETs have been shown to stimulate macrophages to produce high levels of IL-8 and TNF, and induce the differentiation of CD4+ T lymphocytes into IFN-γ producing lymphocytes [150]. However, the data published in the literature remain questionable and depend on the disease model studied. In an acute myocardial infarction model, NETs induced macrophage polarization to the M2 profile, and the deficiency in NETs formation worsened acute inflammation and tissue damage after myocardial infarction, suggesting a protective effect exerted by NETs in this model [151].

Several studies have shown the cooperation between macrophages and neutrophils, and recently, in a thrombosis model in mice, it was observed that even non-polarized macrophages were capable of degrading NETs, but macrophages with a pro-inflammatory profile had a greater degradation capacity [134]. NETs degradation occurred through DNases, and the DNA fragments were internalized via macrophage macropinocytosis. Since NETs work as structures that activate clotting factors, their presence can contribute to thrombus formation. In this regard, the inhibition of macropinocytosis in monocytes led to an increase in NETs load and a reduction in thrombus resolution in vivo [134].

Coronary thrombosis occurs, due to the rupture of the atherosclerotic plaque [147]. NETs and METs are involved in the formation of atherosclerotic plaques and coronary thrombosis in patients who died from acute myocardial infarction, and although other cells have also been related, neutrophil and macrophage ETs were the most frequent [141,147]. NETs predominate in early thrombosis, while METs predominate in chronic thrombosis. METs were more abundant in the intact plaques (lipid core) and in the organized thrombus, since macrophage death contributes to the growth of the lipid core in atherosclerotic plaque [141]. The authors also propose the use of ETs formation as a biomarker for the progression of coronary thrombosis [147]. Activated platelets can also induce ETs formation by macrophages. A study in the murine model of rhabdomyolysis showed that the heme generated by muscle lysis led to platelet activation, which in turn induced the formation of METs, contributing to kidney damage [152]. Another curious fact regarding the cooperation between neutrophils and macrophages is the effect of the proteins present in NETs on the triggering ETs formation in monocytes in vitro [145]. In an experiment designed to show the participation of monocytes in NETs degradation, the exposure to NETs stimulated the formation of extracellular traps by the monocytes themselves. The authors demonstrated that the supernatant of the NETs was capable of stimulating ETs in monocytes, which was caused by citrullinated histones and elastase [145].

Despite all the knowledge already described METs and NETs, it is still unclear whether the interaction between these two effector mechanisms is beneficial or harmful. This is likely to depend on the model of infection or inflammatory disease. This knowledge may be used in new therapeutic or diagnosis/prognosis strategies, as described by Tian et al., who detected CitH3 in the serum of patients with septic shock, and could associate the levels of this compound with the severity and the prognosis of the disease [153].

## 5. Mast Cells

Mast cells (MCs) are derived from the myeloid progenitor in the bone marrow. They circulate in the blood as precursor cells, and when they reach the target tissues, they mature into effector granular cells. They are cells with a monolobulated nucleus, with specific granules and the absence of cytoplasmic glycogen aggregates. Their granules are composed of histamine, heparin, tryptase, and chymase. There are different subclasses of MCs according to the composition of the proteases contained in their granules, morphology, location, and degranulation potential [154,155].

Initially recognized for their role in allergic reactions, it is now widely discussed that long-lived resident MCs are involved in several initial immune responses to various pathogens. Their presence close to the vascular and lymphatic endothelium spreads their products to other locations, enabling MCs to act both locally and remotely [156]. MCs are located at the host-environment interfaces, being abundant in the skin, intestinal mucosa, and respiratory tract, working as sentinel cells. They have direct pivotal microbicidal activity, but can also interact, activate, and recruit other cells to the site of infection through the release of mediators. MCs participate in tissue repair and the regulation of angiogenesis and may influence the progression of tumors and chronic inflammation observed in some types of cancer [157,158].

Several stimuli, such as drugs, food, fungi, viruses, and bacteria, can trigger MC degranulation or activation without degranulation [159]. When stimulated, they present a biphasic response. In an initial phase, they promptly respond to the stimulus through degranulation and the release of preformed inflammatory mediators. Moreover, in a second moment, they secrete de novo synthesized mediators [156]. MCs can secrete β-hexosaminidase, histamine, TNF, tryptase, and prostaglandin D2 within minutes, besides being the only cells capable of storing preformed TNF, making them the first cells to release TNF. After stimulation, they can also secrete cytokines, chemokines, and several growth factors, actively taking part in the initial profile of inflammatory mediators [160,161]. Immune responses to bacteria, viruses, or parasites started by MCs involve different triggering mechanisms and different mediator releases [157,162].

Along with degranulation and mediator release mechanisms, MCs can produce ETs from stimulation with H_2_O_2_, PMA, and various pathogens. ETs in MCs are known as MCETs (mast cell extracellular traps). The presence of MCETs in vitro related to various infections caused by bacteria, protozoa, fungi, and also in other pathological conditions has already been described [163]. MCETs are comprised of nuclear DNA, tryptase, histones, and cathelicidins. Because of the presence of tryptase, both DNase and tryptase-specific proteinases are required for the complete degradation of MCETs [33]. Cathelicidins are antimicrobial peptides (AMPs) that have an antimicrobial effect on bacteria, fungi, enveloped viruses, and protozoa. In humans, cathelicidin LL-37 has already been identified, as well as cathelicidin-related AMP (CRAMP) in mice [164]. Typically, the formation of MCETs is ROS-dependent [163], and MCs undergo nuclear membrane rupture and subsequently cell death [33].

At first, MCETs were observed in a study with *Streptococcus pyogenes*, a bacterium responsible for different human infections, from impetigo to acute necrotizing fasciitis and septic shock. Von Köckritz-Blickwede et al. investigated the in vitro induction of MCETs in response to the human MC (HMC-1) and bone marrow-derived MCs (BMMCs) lines to *S. pyogenes*, and observed a proximity-dependent mechanism, which was not phagocytosis, where MCs were able to inhibit the growth of bacteria. ROS-dependent MCETs induction occurred, since the previous treatment of cultures with NADPH oxidase inhibitor destroyed the antimicrobial effect. Moreover, the addition of DNase and MPO to cultures also destroyed the antimicrobial effect. During MCETs formation, MCs died, due to the rupture of the nuclear membrane, as already described for NETs. The induction of MCETs also occurred in the presence of other human pathogens, such as *Pseudomonas aeruginosa* and *Staphylococcus aureus*. Besides the direct antimicrobial role of MCETs shown by the presence of dead bacteria in MCETs, the authors suggested that such structures could also be useful to restrain harmful substances released by MCs, thus mitigating possible tissue damage [33].

Several studies have corroborated the aforementioned findings demonstrating the importance of MCETs formation by HMC1 and BMMC cells, decreasing the viability of *S. aureus*. The direct microbicidal effect of MCETs associated with the secretion of compounds, such as β-hexosaminidase, tryptase, and TNFα on MC degranulation seems to be an important mechanism for the initial control of *S. aureus* infection. Nevertheless, as an escape mechanism, bacteria are internalized via an active process by MCs and survive in cytosol, which may lead to persistent infection [165]. This escape mechanism was also observed in a study on the cellular mechanisms involved in the pathogenesis of nasal polyps by internalization of *S. aureus*, which allows the survival of bacteria [166]. In vitro, HMC1 cells were able to trap the bacteria within MCETs and then internalize *S. aureus*. The infection would be maintained by cycles of cell disruption, bacterium release, trapping within the MCETs, internalization, and further disruption [166]. The role of hypoxia-inducible factor-1-α (HIF-1α) in MCETs formation in the MC-*S. aureus* interaction was also demonstrated [167]. HIF-1α induces ROS-dependent MCETs formation, for the use of HIF-1α-deficient BMMC antagonists or cells eliminate antimicrobial capacity. The increase in the expression HIF-1α can strengthen the antimicrobial activity, showing its influence on the growth control of *S. aureus*. The authors, therefore, suggest that understanding such interactions may lead to developing new drugs capable of controlling or inhibiting bacterial growth, restraining infections that can reach variable severity, including severe prognosis [167].

Since most of the studies showed that MCETs formation is ROS-dependent and that pathogens can induce different MC responses, Garcia-Rodrigues et al. analyzed the response pattern of human MCs obtained from blood mononuclear cells and differentiated in vitro (hMC) according to DNA and chemokine release, degranulation, and the presence or absence of ROS vis-à-vis pathogens from different tissues. The authors observed that each pathogen-induced a type of MC response [168]. Whereas *L. monocytogenes*-induced degranulation and large DNA release in the absence of ROS, *S. pneumoniae* could not induce degranulation, despite a minimal DNA and ROS release. *E. coli* induced low levels of degranulation with the secretion of IL-8 and MCP-1, with no DNA and ROS release. *S. aureus* induced DNA release and PGD2 secretion by hMCs. These results showed that these MC response mechanisms can be activated independently, as well as that the stimulus has a direct influence on the type of response of hMCs. Based on the results, the authors suggested that hMC cells would present both suicidal and vital MCETs in response to *L. monocytogenes*, being rapid DNA release important for mediator secretion and antimicrobial activity [168]. It had previously been demonstrated that *L. monocytogenes* can induce MCETs formation in a ROS-dependent process with membrane rupture and death of HMC1-dependent, in part based on the release and activity of β-hexosaminidase, as its blockade restored bacterial growth [169]. Opposite results of whether the process is ROS-dependent or not can be caused by the different sources of MCs used in the studies, since in vivo MCs are known to have specific responses according to their granules and tissue location.

MCETs formation has also been described in the group A *Streptococcus* (GAS), gram-positive bacteria capable of producing various infections in humans, ranging from skin infections and pharyngitis to endocarditis and septicemia. An in vitro study on the role of GAS M1 protein in MCETs induction compared wild-type bacteria with mutant bacteria in M1 expression or treatment with purified M1 [170]. The results showed that the expression of this protein played an important role in the induction of MCETs by HMC1. Moreover, it was observed that GAS strains associated with invasive forms of infection were resistant to the antimicrobial effect of cathelicidin LL37 and death by MCETs. The loss of M1 expression was able to confer susceptibility to death by MCETs once again. Thus, the M1 protein strain and the origin of the bacterial isolates could influence resistance to LL37, making certain GAS strains capable of escaping the antimicrobial effect of LL37 and death by MCETs, and as a result, with no control of infection [170]. Moreover, another study demonstrated that MCETs play a fundamental role in the control of GAS infection through changes in the integrity of the membrane produced by LL-37, since the inability to promote MC degranulation during the onset of infection does not allow the control of GAS infection [171].

It has been shown that MCs can control *Enterococcus faecalis* growth via MCETs formation. It was not the only mechanism involved, since, in addition to the evidence of dead bacteria beyond the MCETs, the disruption of these structures partially inhibited growth. Significant degranulation was observed in vitro, suggesting that both; the release of antimicrobial components into the extracellular medium and the induction of MCETs would be important mechanisms to control an *E. faecalis* infection [172].

In vitro studies with Mtb demonstrated that heat-killed Mtb (HK-Mtb) could induce DNA release and that this release also contained tryptase and histones, being consistent with MCETs. This process was H_2_O_2_-dependent, since the inhibition of NADPH oxidase decreased the release of DNA. However, MCETs induced by HK-Mtb and PMA were unable to kill the bacteria. On the other hand, viable Mtb did not produce H_2_O_2_ or induce MCETs. The inhibition of MCETs formation through viable Mtb was related to H_2_O_2_ decomposition by catalase activity in microorganisms. This inhibition would act as a mechanism to evade Mtb from the microbicidal effects of MCETs [169].

MCETs seem to play a role not only in infections caused by bacteria, but also by fungi and protozoa. MCETs formation in the presence of *C. albicans* was observed in vitro, but these structures were not able to decrease the viability of the fungi, which suggests that MCETs formation in *C. albicans* would work as a mechanism of physical restraint of the fungi, so it cannot directly inhibit growth [173]. In *Leishmania* spp., both *L. donovani* and *L. tropica* were able to induce MC death and MCETs formation. The extracellular killing of the parasites in both species was MCETs-dependent, as treatment with DNase increased the viability of promastigotes, both in cultures of peritoneal MCs and in cultures with rat basophilic leukemia (RBL-2H3) mast cell line. The authors suggested that MCETs could be important in the innate immune response formed by MCs towards *Leishmania* spp., since these cells are present in the skin and MCETs can kill promastigotes and arresting them. The signaling mechanisms, as well as the evasion of parasites towards them, might contribute to different outcomes of *Leishmania* spp. infections [174].

All the results discussed refer to in vitro studies, and a direct correlation between these findings and the development of infections caused by these pathogens is not possible. There is still scarce information about the role of MCs and MCETs in vivo. There are three murine models for in vivo studies of MCs: C-kit-dependent MC-deficient mice, c-kit independent MC-deficient mice, and mice with restricted MC mediators. Unfortunately, in humans, most studies use in vitro assessments of human cell lines, such as HMC1 [175]. However, two studies have shown evidence of the possible in vivo role of MCETs. In skin biopsies from patients with psoriasis, cellular expression of interleukin 17 (IL-17) has been demonstrated. This cytokine plays an essential role in the pathogenesis of this disease. Most of the IL17+ cells were MCs, and MCETs formation was observed, especially in normal-looking symptomless psoriatic skin and psoriasis plaques. Besides being more compact than MCETs formed in vitro, in vivo MCETs were a release mechanism for IL-17 by MCs, and were induced by the action of IL-23 and IL-1β. The authors suggested that a possible therapeutic mechanism with targeted drugs for IL-23 might work to decrease NETs and MCETs formations, modulating the effect of these structures on psoriasis lesions [176].

In cardiovascular diseases, the role of NETs in coronary atherosclerosis has already been described [141]. Recognizing that other cell types can form ETs, their role in atherothrombosis was evaluated by immunohistochemistry in coronary plaques from autopsy and in thrombus aspiration samples from patients who died of myocardial infarction. A greater number of ETs were observed in atheromatous plaques that presented thrombotic complications compared to intact plaques, with NETs, METs, MCETs, and EETs (eosinophil extracellular traps) being observed in descending order. Additionally, all types of ETs were also observed in coronary thrombus aspirates, but their presence varied according to the type of cell, as well as the age of the thrombus. Although METs and NETs outweighed MCETs and EETs, MCETs appeared in higher numbers in the organized thrombi. Thus, the authors suggest that ETs formation is involved in thrombus progression and maturation and that MCETs might help destabilize the coronary plaque by releasing anti-inflammatory cytokines and mediators by MCs [141].

MCETs formation seems to be an active process induced or inhibited by different stimuli. It is followed by a series of variations in the production of mediators and ROS, as well as degranulation, and induction or not of cell death (respectively suicidal or vital MCETs). Hence, their role in protecting or worsening a given infection seems to depend on the type of stimulus, the type of MC, and probably in vivo on the type of resident cells or on migration into the site of the infection. The development of conditions for the in vivo study of these cells can provide valuable information for the understanding of MCETs formation. Table 3 shows a summary of the stimuli and types of MCETs observed in vitro and in vivo.

## 6. Eosinophils

Eosinophils are granulocytes derived from the myeloid progenitor in the bone marrow, whose production is regulated by the secretion of hematopoietic growth factors, GM-CSF, IL-3, and IL-5. Although GM-CSF and IL-3 also increase the production of other myeloid cells, IL-5 only increases the production of eosinophils. Under normal conditions, they are cells that are found at low frequency in the blood (1–5% of circulating leukocytes) and other tissues, such as lungs, gastrointestinal tract, thymus, adipose tissue, and in secondary lymphoid organs [178]. Moreover, they have a bilobed nucleus, and cytoplasmic granules that contain primary basic proteins (primary granules) and eosinophilic cationic proteins (secondary granules) that are toxic to various parasites and mammalian cells. The primary granules comprise Charcot-Leyden crystal protein, also known as galectin 10, and eosinophil peroxidase (EPO), while the secondary granules contain, in addition to EPO, major basic protein (MBP), eosinophilic cationic protein (ECP), and eosinophil-derived neurotoxin (EDN) [178].

Typically, the increase of eosinophils in the blood or the presence of eosinophilic infiltrate in tissues is observed in allergic reactions, such as asthma and chronic rhinosinusitis, in helminth infections, and also in some bacterial and fungal infections [179,180]. Immunoregulatory actions for eosinophils, such as lymphocyte recruitment and tissue repair, have also been described [181].

Unlike neutrophils, eosinophils are not phagocytic cells, performing their defensive activity by the selective release of granular content into the extracellular environment. During degranulation, EPO, which differs significantly from the peroxidase in other granulocytes, interacts with H_2_O_2_, generating cytotoxic oxygen radicals for tumor cells, HIV, and schistosomula of *Schistosoma mansoni*. EDN has ribonuclease activity that acts against single-stranded RNA viruses, such as HIV and respiratory syncytial viruses, while ECP has antiparasitic and antibacterial activities. MBP-1 is toxic to bacteria, schistosomula of *S. mansoni*, and can injure host tissues with eosinophilic infiltrate. Moreover, MBP-1 has immunoregulatory activity, such as an increase in the pro-inflammatory cytokine IL-8. Charcot-Leyden crystals are essentially composed of phospholipase B and are found in phlegm, tissues, and feces in diseases that have an intense inflammatory response, indirectly evidencing the release of eosinophil granules (reviewed by the authors of [182]).

In 2008, Yousefi et al. reported that degranulation was not the only way eosinophils acted, demonstrating both ex vivo (using colon biopsy from patients with schistosomiasis, Crohn’s disease, or intestinal spirochetes), as well as in vitrostudies on colocalization, the presence of EETs, and that the DNA present was mtDNA, with eosinophil granule proteins, such as MBP and ECP, incorporated into the multiple extracellular DNA fibers observed. The reaction depended on the activation of NADPH oxidase and release of ROS. Eosinophils remained viable during the process (vital EETs) [183]. Stimulation of human eosinophils with thymic stromal lymphopoietin also induced the release of mitochondrial-originated EETs [184].

Subsequently, a process of release of EETs with nuclear origin occurring with cell death was described, which was initially called EETosis (as the mechanism observed for neutrophils) [185]. In this study, EETs released by human eosinophils were observed after in vitro stimulation with immobilized immunoglobulins (IgG and IgA), platelet-activating factor (PAF), calcium ionophore, or PMA. Eosinophil cytolysis (suicidal EETs) was observed, and EETs were composed of nuclear DNA associated with histones and eosinophil granules. The process was also NADPH oxidase-dependent [185].

The production of vital EETs (mtDNA) or suicidal EETs has been associated with allergic eosinophilic diseases, such as allergic asthma, rhinosinusitis with nasal polyps, eosinophilic esophagitis, chronic obstructive pulmonary disease, allergic bronchopulmonary aspergillosis, and eosinophilic otitis media [186,187,188,189,190]. Recently, suicidal EETs formation by murine and human eosinophils has been observed in the presence of microfilariae and infective L3 larvae of *Litomosoides sigmodontis* and microfilariae of *Dirofilaria immitis*, in a Dectin-1-dependent manner [191].

In eosinophilic esophagitis, EETs formation with mtDNA was correlated with the number of eosinophils in the tissue. An inverse correlation of the serine protease inhibitor protein LEKTI with a number of EETs suggested a possible protective role of eosinophils against invading pathogens, regarding disruption of the epithelial barrier, where EETs would work as a secondary barrier [186].

Airway inflammation resulting from eosinophilia is closely related to Severe Eosinophilic Asthma (SEA). The high production of granular proteins, such as ECP, EDN, and MBP, identified in patients with SEA indicates activation and degranulation of eosinophils. Moreover, patients with asthma, chronic lung diseases, and viral respiratory infections produce large amounts of IL-8 at inflammatory sites, a cytokine closely related to EETs production. The high level of eosinophil activation observed in SEA leads to an increase in ROS production and EETs formation, resulting in inflammation and airway obstruction in patients with SEA, in a NADPH oxidase-dependent manner [188]. In a murine model of acute asthma, EETs have been shown to increase mucin secretion in the airways of animals after the OVA challenge [192]. Controlling EETs formation and its activity may provide innovative treatment methods for patients with asthma [192,193].

The correlation between the viscosity of eosinophil-rich exudates and EETs formation has been demonstrated microscopically in secretions obtained from patients with chronic eosinophilic rhinosinusitis (ECRS) and eosinophilic otitis media (EOM) [185,187,190,194]. The authors demonstrated that EETs were composed of thick fibers associated with eosinophil granules and H1 histone, indicating nuclear DNA with cell death (suicidal EETs). Regarding ECRS associated with *S. aureus*, it has been suggested that eosinophils are likely to be specifically recruited for *S. aureus* and possibly for other microorganisms, thus forming EETs at epithelial damage sites to protect the host from infection [195].

Allergic bronchopulmonary aspergillosis (ABPA) affects asthmatic patients and individuals with cystic fibrosis in response to several antigens of *A. fumigatus*, which colonize the bronchial mucus. The assessment of mucus, obtained from the airways of patients with ABPA, showed suicidal EETs formation, with citrullinated histone 3 and intact eosinophil granules [189,196]. Eosinophils stimulated in vitro with *A. fumigatus* antigens did not induce ROS production, since inhibition of NADPH oxidase activity or mtROS generation did not inhibit EETs formation. However, it is dependent on the pathway of CD11b and Syk tyrosine kinase. Interestingly, these fungus-stimulated EETs did not show fungicidal or fungistatic activity towards *A. fumigatus* [189]. In a recent study on the characterization of the mechanisms involved in EETs formation in ABPA, the dependence on the signaling pathways p38 MAPK, Akt, Src, calcium, and PI3 was demonstrated, regardless of the viability of the fungus. Remarkably, the release of EETs was independent of histone citrullination by PAD-4 [197]. In concert, the results suggest that EETs may be produced by several pathways in response to antigenic stimuli.

EETs have also been identified in non-allergic inflammatory processes, such as sepsis and colitis [183], atherosclerotic plaque formation, and thrombosis [141,198]. In atherothrombosis, eosinophils form ETs after interacting with platelets, and eosinophils participate in platelet activation. The formed EETs comprise a significant part of the DNA traps found in human and murine thrombi, presenting a large amount of main basic protein (MBP) adhered to DNA filaments [198]. Moreover, the origin of DNA (mitochondrial or nuclear) has not been evaluated.

EETs formation has also been shown in some atopic dermatitis [199], such as bullous delayed pressure urticaria lesions, where EETs formation seems to be related to the apoptosis in keratinocytes and blister formation. However, the mechanisms involved in EETs formation and function have not yet been elucidated [199,200,201].

Despite the need for more information, the study on EETs induction mechanisms in eosinophilic diseases, whether allergic or not, or in autoimmune and cardiovascular diseases, has received a great deal of attention in the last decade because it may bring new alternatives to treat these diseases. Table 4 shows a summary of EETs and their possible roles in eosinophilic, autoimmune, and cardiovascular diseases discussed in this document.

## 7. Lymphocytes

T and B lymphocytes result from stimulation of lymphoid progenitors in the bone marrow, and the selection and clonal maturation of which occurs in the bone marrow (B lymphocytes) or in the thymus (T lymphocytes). They are mononuclear cells known as the major orchestrators of the immune response, as they participate in both, stimulation/regulation and in the effector function of the inflammatory process. In short, lymphocytes are involved in the presentation of antigens via the major histocompatibility complex (MHC) class II (B lymphocytes), cytokine production (B and T lymphocytes), a stimulus to the effector phase of other lymphocytes, as well as other cells associated with immune response, such as macrophages and granulocytes. They are also capable of exerting cytotoxicity on target cells by MHC class I recognition followed by direct degranulation, or by receptor-ligand binding, such as Fas-FasL, TRAIL, and others. Besides, they can form and maintain immune memory, and act in both humoral (B lymphocytes) and cellular (T lymphocytes) immune responses. T lymphocytes are now considered as central cells in the organization, targeting, and modulation of inflammation. There has not been enough evidence in the last few years that lymphocytes might produce ETs. Many of the results were obtained in vitro, but some direct or indirect evidence of their in vivo role has been identified. Nevertheless, many questions need to be clarified, although the first results point to the possibility that DNA extravasation plays a role in infectious and non-infectious diseases, especially in autoimmune diseases. According to the authors, the phenomenon has been named differently, but the term lymphocyte-derived extracellular traps (LETs) has now been used, and will, therefore, be referred to in this document [32].

To our knowledge, the first evidence of the participation of lymphocytes in ETs formation emerged in 2017 [202], when the formation of extracellular structures rich in DNA from B lymphocytes stimulated with PMA, ionomycin, anti-IgM, LPS, or serum from patients with SLE, as well as with serum from other types of autoimmune diseases, such as cryoglobulinemic vasculitis and Sjögren’s syndrome, all characterized by the formation of immune complexes, was verified in vitro. In the same study, other autoimmune diseases, such as RA and dermatomyositis, did not have a similar effect on B cells. The authors were able to detect plasma membrane damage in B lymphocytes. In this paper, similar data were identified in T cells. Nevertheless, the authors did not evaluate other protein molecules associated with extravasated DNA, although the data suggest ETs formation and B lymphocyte death by ETosis [202].

In 2018, Ingelssom et al. demonstrated the extracellular release of mtDNA by B lymphocytes. mtDNA is known to be rich in CpG motifs, which are recognized by TLR-9 [14]. Stimulation by oligodeoxynucleotides with or without CpG is observed in cells, such as B lymphocytes, T lymphocytes, NK, neutrophils, and macrophages. The authors described that the formation of filaments was different from that observed in neutrophils, and their presence was not stimulated by lipopolysaccharides (LPS) or PMA. B cells showed the release of long filaments produced independently of BCR, ROS, and without evidence of cell death. Due to the absence of toxic proteins coupled to DNA filaments, the authors suggested that this type of DNA extravasation would work as a DAMP, associated with the triggering of an innate immune response and stimulating the production of IFN-1 [14].

The characterization of LETs formation in activated T lymphocytes was subsequently published for both CD4+ T cells [32,203] and CD8+ T cells [32], and with this description of LETs formation, another function was associated with a plethora of important functions of lymphocytes.

Costanza et al. demonstrated the presence of LETs in CD4+ cells both in vitro from cell stimulation with anti-CD3/anti-CD28, and in the experimental model of autoimmune encephalomyelitis (EAE) [203]. In vitro, DNA expression was verified with an association of histones and identification of damage to the plasma membrane, and the formation of several filaments involving activated CD4+ T lymphocytes connected to the adjacent lymphocytes. The extracellular DNA was destroyed by DNase, and resting CD4+ T lymphocytes were weakly positive in the stains performed, evidencing activation as a key orchestrator for DNA release. The phenomenon also seems to be governed by ROS, since its inhibition reduces DNA release without altering cell activation and proliferation. It also showed an increase in the production of IL-2, GM-CSF, IFN-γ, and TNF-α in CD4+ cell cultures with LETs, suggesting that LETs formation might work as a second signal for effective action upon lymphocytes. Interestingly, tagged mtDNA showed that it was part of the LETs produced by CD4+ cells [203]. The authors identified that the inhibition of mtROS entailed a decrease in lymphocyte activation in vitro, cytokine production, and LETs formation. In vivo, the cells maintained the proliferation capacity, but not the cytokine production. The presence of LETs containing DNA and histones was detected in CD4+ cells present in lymph nodes of mice with EAE. The inhibition of LETs formation caused an improvement in the condition of EAE, which evidences the implication of LETs in the pathogenesis of the disease. Due to the concurrent presence of histones and mtDNA, the authors were unable to determine whether the phenomenon in CD4+ lymphocytes was suicidal or vital [203]. The presence of a combination of mtDNA and DNA containing histone in other cell types, such as neutrophils, has already been described [25]. Moreover, as LETs formation is quite fast, the results on various cell types published so far suggest that the phenomenon might occur simultaneously or be organized as a sequence of temporal events, due to the evolution of cellular structures, since damage to the plasma membrane is evident.

Koh et al. has recently identified that after in vitro stimulation with anti-CD4/anti-CD28, CD4+ cells produce diffuse ETs that surround the cell like a halo and with evidence of cell death, and that this formation differs from ETs in CD8+ T lymphocytes, whose ETs form filaments [32].

In addition to cytokine production, CD8+ cells feature as one of the main effector functions of direct cytotoxicity (CTL) from the T-cell receptor (TCR) recognition of MHC class I molecules containing antigen on the surface of infected/altered cells, called target cells, with the association of costimulatory molecules. Subsequently, the granular content is released into the area of contact between the cells and the action of enzymes in a cascade of events that result in nuclear DNA degradation and the rupture of the plasma membrane, leading to cell destruction. This classic mechanism of CTL was added to the description of ETs production by CD8+ T lymphocytes by Koh et al., who evaluated LETs formation by CD8+ T lymphocytes both in vitro by stimulation with anti-CD3/anti-CD28 and in vivo in ATL lesions [32]. The authors detected the formation of long filaments of extracellular DNA containing enzymes of granular content shown by the colocalization of CD107a, resulting in cell death by LETs. Electron microscopy confirmed ETs of CD8+ T lymphocytes via disruption of the cell membrane and polarization of organelles. This action was also verified ex vivo by the in situ study on ATL lesions of various clinical presentations. The comparative evaluation showed that the presence of LETs was associated with greater severity of the lesions, leading to a correlation between a higher concentration of CD8+ cells forming LETs and the most exuberant inflammatory conditions in ATL. The in vitro study also identified the association of LETs formation with an increase in intracellular Ca++ and the absence of association with NOS2 and ROS, showing particular features of extracellular DNA formation and release structure in CD8+ cells when compared to other cell types [32].

Unlike the CTL mechanism, LETs formation can reach cells at a distance, increasing the action capacity of CD8+ T cells. Furthermore, disruption of both the target cell and the CD8+ cell leads to the release of intracytoplasmic content and can cause additional inflammatory stimulation [32].

Other studies should be performed to elucidate the unclear points concerning LETs formation by T and B lymphocytes and their action on the inflammatory process of different etiologies. The results published so far point out that this possibility is quite robust, and this intensity and regulation role of the immune response should be considered. Clarifying whether they play a role in the protection and/or exacerbation of different diseases may bring subsidies to the design and production of therapeutic targets able to modulate the immune response and consequently tissue damage caused by inflammatory phenomena. Table 5 shows a summary of the data discussed concerning LETs formation and function.

## 8. Other Cells Involved in the Immune Response Whereby the Formation of Extracellular Traps Has Been Identified

Basophils and plasmacytoid dendritic cells have been identified as capable of producing ETs, requiring a greater understanding of the formation, stimuli, and application in disease or protection during the inflammatory process. The information regarding the ETs produced by these cell types is shown in Table 6.

### 8.1. Basophils

Basophils are scarce blood leukocytes (about 2%), produced in the bone marrow from myeloid progenitors. Their granules are metachromatic, larger than other granulocytes, and contain hydrolytic enzymes, chemotactic factors for neutrophils and eosinophils, heparin, and histamine. They have receptors on the plasma membrane to bind to immunoglobulin E (IgE), which after subsequent exposure to the allergen, release their granules, leading to vascular disorders associated with hypersensitivity and anaphylaxis [208].

Besides, basophils can produce ETs within a few minutes of stimulation by IgE, chemokines, TLRs, cytokines, and lipid mediators. Basophil extracellular traps (BETs) are formed from mitochondria and by a mtROS-dependent and NADPH oxidase-independent mechanism [204,205]. However, there is still little information about the mechanisms and implications of this function in basophils. Considering that they are not capable of intracellular killing of bacteria like neutrophils, basophils can trap and kill microorganisms through BETs, as already demonstrated against *E. coli* and *S. aureus* [208]. The development of methodologies to study these cells will enable us to obtain data that may provide a better understanding of the action of basophils upon inflammatory processes, including the participation of BETs.

### 8.2. Plamacytoid Dendritic Cells

Dendritic cells (DCs) are bone marrow-derived from pluripotent hematopoietic stem cells considered one of the major antigen-presenting cells of the immune system. Although they account for less than 1% of leukocytes in peripheral blood, these cells are located in other tissues where they act as sentinels of the immune system, patrolling the presence of antigens for presentation to T lymphocytes, playing a critical role in linking innate and adaptive immune responses. They are classified into subpopulations according to location, function, etc. Recently, an article identified in vitro ETs formation derived from plasmacytoid dendritic cells after recognizing hyphae of *A. fumigatus*. The recognition was Dectin-2-dependent and led to the ETs formation comprised of nuclear DNA and citrullinated histone H3 [207]. Further studies should be conducted to elucidate the mechanisms and participation of dendritic cell extracellular traps (DCETs) in the inflammatory process.

## 9. Concluding Remarks

All these years of studies and data collection on ETs identify a variety of cell types involved, as well as formation mechanisms and potential actions of releasing extracellular traps upon protection and disease. However, information capable of elucidating some important mechanisms is still deficient. While the mechanisms involved have been formerly associated only with the innate immune response, nowadays, their involvement in a specific immune response are discussed—mainly at the expense of recent descriptions of ETs in lymphocytes. Moreover, considering that there are not enough in vivo studies, it is essential to develop experimental models and studies on human biological material that can verify the influence of ETs on the pathophysiology of infectious and non-infectious diseases, such as autoimmune diseases. Much is already known, but much more needs to be known to understand the dynamics of ETs in inflammation. For instance, how the cell is stimulated to develop ETs and not another effector mechanism, the effect of these traps on the infectious/inflammatory process, and the impact of these extracellular traps on the other types of cells involved in the immune response. On the other hand, there is no doubt that all the knowledge generated so far regarding the formation of ETs. Many data that are generated daily on this subject, point to the possibility of developing new drugs, or repositioning already known drugs, capable of preventing the development of ETs or leading to their dissolution, as immunotherapeutic alternatives in a variety of infectious and non-infectious diseases, notably those with immunothrombotic characteristics, such as COVID-19. The immune system sets its traps; however, we still do not fully understand how and what the consequences of this movement are.

## Figures and Tables

**Table 1:** Neutrophil extracellular traps in host defense and disease.

Cell	Mechanism of ETs Formation	Stimulus/Models	Biological Effect Protective Deleterious	
Neutrophil in Cancer	Suicidal (ROS-dependent) [91,107,108,109,110] Early/rapid ROS-independent (but may alternatively be dependent on autophagy) [111] Mitochondrial NETs [61]	In vivo Murine models of: breast cancer [91], lung carcinoma [107], metastatic colorectal cancer [109,110], lung carcinoma [108] Ex vivo Serum samples of patients with metastatic colorectal [109,110] and human tissue samples of breast cancer [91] In vitro Cancer cells [91], pancreatic cancer cells [111], anaplastic thyroid cancer cells [61]	Entrapment of tumor cells [107]	Association with an aggressive subtype of breast cancer [91] Tumor progression [61,110] Metastasis [91,107,108,109,110] Reduction in disease-free survival [109] Cancer-associated thrombosis [111]
Neutrophil in Central Nervous System Diseases	ROS-dependent [112] Nuclear DNA [113,114,115,116]	In vivo Murine model of Alzheimer’s disease, meningitis and [112,116] Piglet model of S. suis meningitis [113] In vitro Thrombi from patients with acute ischemic stroke [114,115]; paraffin sections of human cortex from Alzheimer’s disease brains [116] CSF of patients with S. pneumoniae meningitis [112] Modified human BCSFB model [113]	Entrapment of streptococci [113]	Alzheimer’s disease pathogenesis [116] Impairment of pneumococci clearance in meningitis [112] Poorer clinical outcomes and inflammation aggravation in patients with acute ischemic stroke [115]; Important constituents of cerebral thrombi [114]
Neutrophil in Pulmonary Diseases	Suicidal, ROS-dependent [117,118] ROS-dependent [119] Nuclear DNA [120,121,122]	In vivo Murine and human model of rhinovirus-induced allergic asthma exacerbation [122], murine model of S. pneumoniae induced pneumonia [119], and PTB [121] Ex vivo Human lung samples [121] In vitro Sputum samples of asthma patients/human airway epithelial cells [117] Sputum samples of COPD patients [118,120]		Asthma severity and exacerbation [117,122] Airway epithelial and endothelial damage [117] Severity of S. pneumoniae induced pneumonia [119] COPD severity and airway flow limitation [118,120] PTB pathogenesis and severity [121]
Neutrophil in Autoimmune Diseases	ROS-dependent [123] Mitochondrial NETs (mtDNA, mtROS) [25] Not described [15]	In vitro Immune complexes (Anti-LL-37, anti-HNP, PR3 and MPO, ANCAs) [123] Healthy and lupus neutrophils (PMA and immune complexes) [25] Healthy and rheumatoid arthritis neutrophils (PMA and A23187) [15]		Autoimmune diseases (systemic lupus erythematosus, psoriasis, vasculitis, rheumatoid arthritis) [15,25,123,124]
Neutrophil in Thrombosis/Cardiovascular Disorders	Nuclear DNA [125] ROS-dependent [126]	In vitro Blood neutrophils and platelets [125] In vivo Deep vein thrombosis model (Baboons) [125] In vivo Murine model (cholesterol crystals) [126]		Thrombosis [125] Atherosclerosis [126]
Neutrophil and Virus	ROS-dependent [127,128] Suicidal, ROS-dependent [92] PAD-4 dependent [94] Suicidal, presence of Cit-H3 and MPO-DNA complexes [94,95,96,97,98,99]	In vivo Murine model of influenza A virus H1N1pneumonia [127] and Chikungunya virus infection [128] In vitro Neutrophils + influenza virus–primed epithelial cells [127] Serum samples and/or nasal swab specimens from COVID-19 patients [92,93,94,95,96,97,98,99] Neutrophils + SARS-CoV-2 [92,94] Neutrophils + Chikungunya virus [128] Ex vivo BALF and lung autopsies from COVID-19 patients [94,95,96]	Virus capture, Neutralization and reduction of viral load in the blood. [128]	Lung injury [127] Thrombosis formation in COVID-19 [92,96,99] COVID-19 Pneumonia [97] COVID-19 severity and vascular damage [94,95,98,99]
Neutrophil and Fungi	Suicidal, ROS-dependent [66,129,130] Vital NETs, ROS-independent [65] Not described [30]	In vivo Murine model of A. fumigatus [66] Murine model of *C. albicans* infection [129] In vitro A fumigatus conidia [130] *C. albicans* (β-glucan) [65] Ex vivo Active sporotrichosis lesion [30]	Entrapment of conidia, the only fungistatic effect [66,130] Capture and kill *C. albicans* yeast and hyphal forms [65,129] Antimicrobial effect [30]	
Neutrophil and Protozoa	Early/rapid, ROS-independent, and late ROS-dependent [68] Suicidal, ROS-dependent [64] ROS-dependent [28,73,131] ROS-independent [132] Not described [27,29,67]	In vivo Murine model of *T. cruzi* [131] Murine model of Malaria with *P. berghei* [132] and *P. chabaudi* [73] Murine model of T. gondii [28] Ex vivo ATL active cutaneous lesions [29] In vitro *Leishmania* spp.—amastigotes, promastigote/lipophosphoglycan [64,67,68] *T. cruzi* [131] Blood samples from patients infected with *P. falciparum* [73,132]	Containment of promastigotes at the inoculation site and Leishmania killing [64,68] Limits infection by affecting the parasite’s pathogenicity [131] Antimicrobial effect [29,73,132] Interferes with the parasite’s ability to invade cells [28]	Activation of emergency granulopoiesis via GM-CSF production, and induction of the endothelial cytoadhesion receptor ICAM-1 [73] Stimulus of ANA production, which may lead to autoimmunity [27]

ETs, extracellular traps; ROS, reactive oxygen species; NETs, neutrophil extracellular traps; CSF, cerebrospinal fluid; BCSFB, blood-cerebrospinal fluid barrier; PTB, pulmonary tuberculosis; COPD, chronic obstructive pulmonary disease; mtDNA, mitochondrial DNA; GM-CSF, granulocyte macrophage colony-stimulating factor; CF5a, complement factor 5a; LPS, lipopolysaccharide; TLR4, toll like receptor 4; anti-LL-37, antimicrobial peptide, anti-HNP, human neutrophil peptide; PR3, proteinase-3; BALF, bronchoalveolar lavage fluid; Cit-H3, citrullinated histone H3; MPO, myeloperoxidase; ANCAs, anti-neutrophil cytoplasmic antibodies; PMA, phorbol-12-myristate-13-acetate; oxLDL, oxidized low-density lipoprotein; ICAM-1, intercellular Adhesion Molecule 1; ANA, antinuclear Antibodies; ATL, American Tegumentary Leishmaniasis.

**Table 2:** Macrophage extracellular traps in host defense and disease.

Cell	Mechanism of ETs Formation	Stimulus/Models	Biological Effect	
			Protective	Deleterious
Monocytes/Macrophages and Fungi	Not described [137] ROS and NADPH oxidase-independent manner, mtDNA only or mtDNA and nuclear DNA [136]	In vitro *C. albicans* [136,137]	*C albicans* load control in vitro [137] Entrapment of *C. albicans* [136,137]	
Monocytes/Macrophages and Bacteria	mtDNA only or mtDNA and nuclear DNA, ROS, and NADPH oxidase-independent manner [136] Not described [144] Elastase activity and *M. tuberculosis* ESX-1 [144]	In vitro *E. coli* [136,142] *M tuberculosis* [144] IFN-γ [144] Ex vivo *U. urealyticum* and *C. trachomatis* [142]	*E. coli* load control in vitro [136] Entrapment of *E. coli* and *M. tuberculosis* [136,142,144]	
Monocytes/Macrophages and Protozoa	MPO, ROS, and NADPH oxidase-dependent manner [139,140]	In vitro *E. ninakohlyakimovae* [140] *B. besnoiti*/*E. bovis* [139]	Entrapment of *E. ninakohlyakimova, B besnoiti/E bovis* [139,140]	
Monocytes/Macrophages in Diabetes and Obesity	PAD2/PAD4 mediated histone hypercitrulination [138] Not described [143]	In vitro TNF [138] Not described [143]		Induction of inflammation and insulin resistance [143] Acceleration of inflammation associated with obesity [138]
Monocytes/Macrophages in Thrombosis	Not described [141,147]	Not described [141,147]		Arteriosclerotic plaques and coronary thrombosis formation [141,147] Thrombus instability [147]

ETs, extracellular traps; ROS, reactive oxygen species; NADPH, nicotinamide adenine dinucleotide phosphate mtDNA, mitochondrial DNA; METs, macrophage extracellular traps; ESX-1, ESAT-6 secretion system 1; IFN-γ, interferon gamma; MPO, myeloperoxidase; PAD2, peptidyl arginine deiminase 2; PAD4, peptidyl arginine deiminase 4; TNF, tumor necrosis factor; PMA, phorbol-12-myristate-13-acetate; —HOCl, hypochlorous acid, IL-8, interleukin.

**Table 3:** Mast cell extracellular traps in host defense and disease.

Cell	Mechanism of ETs Formation	Stimulus/Models	Biological Effect	
			Protective	Deleterious
Mast cell and Bacteria	ROS-dependent [33,167,169,177] Suicidal MCETs [33,166] Not described, probably suicidal because DNA released was linked to dead cell staining or nuclear changes were observed [165,167,177] Not described [33,169,170,171,172] Suicidal and vital MCETs, ROS-independent [168]	In vitro HMC1 and BMMC lines + *S. pyogenes/S. aureus/P. aeruginosa* [33] HMC1 + GAS/Purified M1 GAS protein/*L. lactis* [170] HMC1 and BMMC lines + *S.aureus* [165,167] HMC-1 and BMMC lines + *E. faecalis* [172] HMC1 line + *L. monocytogenes* [177] HMC-1 and BMMC lines + Mtb (viable and HK-Mtb)/*S.aureus* [169] HMC-1 and BMMC lines + GAS/*L.lactis*/*S.aureus* [171] HMC-1 + *S.aureus* [166] HMC-1 + *L.monocytogenes*/*E. coli*/*S.aureus*/*S. pneumoniae* [168]	Antimicrobial effect [33,165,167,168,171,172,177]	M1 GAS protein contributes to GAS survival—invasive forms of infection [170] Mtb inhibit MCET formation—bacteria survival [169] Capture, phagocytosis, maintenance of infection [166]
Mast cell and Fungi	Not described, probably suicidal, but dead MC numbers were higher than MCETs observed [173]	In vitro HMC1 + *C. albicans* [173]	Physical restraint only [173]	
Mast cell and Protozoa	Suicidal MCETs ROS-dependent [174]	In vitro RBL MC line + *L. donovani*/*L. tropica* [174]	Antimicrobial effect [174]	
Mast cell and Psoriasis	Not described, probably suicidal because it was observed that MCs were not intact in lesions [176]	Ex vivo MCs from psoriasis lesions [176]		IL-17 release, leading to pathogenic effect [176]
Mast cell And Atherothrombosis	Not described [141]	Ex vivo MCs from coronary plaques and thrombus [141]		Thrombus progression and maturation [141]

Mtb, mycobacterium tuberculosis; ROS, reactive oxygen species; MCETs, mast cell extracellular traps; MC, mast cell; PMA, phorbol myristate acetate; HMC1, human MC line; BMMC, bone marrow–derived MC; RBL, rat basophilic leukemia mast cell line; HK-Mtb, heat-killed Mtb; GAS, group A *streptococcus*; IL-17, interleukin 1.

**Table 4:** Eosinophil extracellular traps in host defense and disease.

Cell	Mechanism of ETs Formation	Stimulus/Models	Biological Effect	
			Protective	Deleterious
Eosinophil in Intestinal (Colon) Diseases	Vital (mtDNA) ROS-dependent [183]	Ex vivo Colon Biopsies from Crohn’s disease, schistosomiasis, and intestinal spirochetosis patients	Entrapment of bacteria [183]	
Eosinophil In vitro (Human PBMC)	Vital (mtDNA) ROS-dependent [183] NADPH oxidase-dependent [184,188] Suicidal (Nuclear DNA) dependent of histone citrullination, CD11b, and the Syk tyrosine kinase pathway [185,187,189] Suicidal-independent of PAD4 histone citrullination and depends on the Src family, Akt, Ca, and p38 MAPK signaling pathways [197]	LPS, C5a, cotaxin/CCL11 [183] Opsonized *E. coli* [183] *A. fumigatus* [189,197] Thymic stromal lymphopoietin [184] Immobilized immunoglobulins (IgG, IgA), cytokines with PAF, Ca ionophore, or PMA [185,187] IL-5 and LPS [188]	Bactericidal activity [183] Entrapment of fungi [197]	Airway inflammation and obstruction in Asthma [188]
Eosinophils in Eosinophilic Diseases	Suicidal (Nuclear DNA) [187,194] Not described [186,190,195,202]	Ex vivo Secretions and tissue slides ECRS patients [187,190,195] Secretions from EOM patients Tissue slides [187,194] Biopsies from EOE patients [186] Skin biopsy tissues of 25 different eosinophilic skin diseases [202]	Firewall against the invasion of pathogens [186,195]	Increase in secretion viscosity [187,194] Inflammation [202]
Eosinophils in Allergic Bronchopulmonary Diseases	Suicidal (Nuclear DNA) [189,196] Dependent of histone citrullination, CD11b, and the Syk tyrosine kinase pathway [189] Not described [193]	Ex vivo BALF [196] Bronchial mucus plugs [189] In vivo Murine animal model of Asthma [193]		Increase in secretion viscosity [189,196] Asthma exacerbation [193]
Eosinophils in Atherothrombosis	Suicidal (Nuclear DNA) [141,198]	In vivo Murine model [198] Ex vivo Human autopsy [141]		Thrombus formation [141,198]

mtDNA, mitochondrial DNA; BALF, bronchial lavage fluid; C5a, complement component C5a; CCL11, C-C motif chemokine ligand 11; ECRS, eosinophilic chronic rhinosinusitis; EOE, eosinophilic esophagitis; EOM, eosinophilic otitis media; ETs, extracellular traps; LPS, lipopolysaccharides; PAD4, protein arginine deiminase 4; PAF, *platelet*-activating factor; PBMC, peripheral blood mononuclear cells; PMA, phorbol myristate acetate; ROS, reactive oxygen species.

**Table 5:** Lymphocyte extracellular traps in host defense and disease.

Cell	Mechanism of ETs Formation	Stimulus/Models	Biological Effect	
			Protective	Deleterious
B lymphocytes	Not described, probably suicidal, since membrane damage is described [202] Vital [14]	In vitro PMA, ionomycin,, anti-IgM,, LPS, SLE serum [202] CPG motifs [14]		Probably autoimmune diseases, SLE, cryoglobulemic vasculitis, and Sjögren syndrome [202] Autoimmune diseases [14]
CD4 T lymphocytes		In vitro antiCD3/antiCD28 [203] antiCD4/antiCD28 [32] In vivo Experimental model of encephalomyelitis [203]		Autoimmune diseases [203] American Tegumentary Leishmaniasis [32]
CD8 T lymphocytes	Suicidal [32]	In vitro antiCD3/antiCD28 [32] Ex vivo American Tegumentary Leishmaniasis lesions [32]		American Tegumentary Leishmaniasis [32]

ETs, extracellular traps; PMA, phorbol-12-myristate-13-acetate; anti-IgM, anti-immunoglobulin M; LPS, lipopolysaccharide; SLE, systemic lupus erythematosus; mtDNA, mitochondrial DNA.

**Table 6:** Basophils and plasmacytoid dendritic cell extracellular traps in host defense and disease.

Cell	Mechanism of ETs Formation	Stimulus/Models	Biological Effect	
			Protective	Deleterious
Basophils	Vital (mtDNA), NADPH oxidase independent [204] Not described [205,206]	In vitro (human blood) Monosodium urate [205] *Staphylococcus aureus* [206] In vitro (human blood and murine Hoxb8-immortalized myeloid progenitors derived basophils) IL-3 priming and subsequent activation of the C5a receptor or FcεRI [204]	Bactericidal activity [206]	
Plasmacytoid dendritic cells	Suicidal (Nuclear DNA) Citrullinated histone H3 Dectin-2-dependent [207]	In vitro (Human PBMC) *Aspergillus fumigatus* [207]	Antifungal activity [207]	

mtDNA, mitochondrial DNA; C5a, complement component C5a; ETs, extracellular Traps; PBMC, peripheral blood mononuclear cells.
